# Translational regulation in response to stress in Saccharomyces cerevisiae

**DOI:** 10.1002/yea.3349

**Published:** 2018-09-03

**Authors:** Robert A. Crawford, Graham D. Pavitt

**Affiliations:** ^1^ Division of Molecular and Cellular Function, School of Biological Sciences, Faculty of Biology, Medicine and Health, Manchester Academic Health Science Centre The University of Manchester Michael Smith Building, Dover Street Manchester M13 9PT UK

**Keywords:** translational control, RNA‐binding proteins, eIF2 phosphorylation, ribosome filter, specialized ribosomes

## Abstract

The budding yeast Saccharomyces cerevisiae must dynamically alter the composition of its proteome in order to respond to diverse stresses. The reprogramming of gene expression during stress typically involves initial global repression of protein synthesis, accompanied by the activation of stress‐responsive mRNAs through both translational and transcriptional responses. The ability of specific mRNAs to counter the global translational repression is therefore crucial to the overall response to stress. Here we summarize the major repressive mechanisms and discuss mechanisms of translational activation in response to different stresses in S. cerevisiae. Taken together, a wide range of studies indicate that multiple elements act in concert to bring about appropriate translational responses. These include regulatory elements within mRNAs, altered mRNA interactions with RNA‐binding proteins and the specialization of ribosomes that each contribute towards regulating protein expression to suit the changing environmental conditions.

## 1 DYNAMIC REGULATION OF PROTEIN SYNTHESIS IN YEAST

### 1.1 The importance of regulating protein synthesis

Yeasts such as Saccharomyces cerevisiae, as single‐celled, non‐motile organisms, must adapt biochemically to survive in rapidly changing external environments (Simpson & Ashe, 2012). Adaptation of the proteome is crucial to respond to the constraints and challenges imposed by cellular stress conditions. For example, amino acid starvation results in a reduction in the level of charged aminoacyl‐tRNAs, limiting the rate of protein synthesis until cells respond. Oxidative stress similarly constrains biosynthetic processes, while also bringing about damage to existing proteins, which must be resolved (Costa, Quintanilha, & Moradas‐Ferreira, 2007). Overcoming such difficulties is one important reason why protein synthesis must be tightly and dynamically regulated.

During diverse stresses significant alterations to the proteome must be made to enable cells to adapt to the changing conditions. S. cerevisiae contains around 6000 open reading frames (ORFs), but only a subset of these are expressed at any one time (Ghaemmaghami et al., 2003; Kulak, Pichler, Paron, Nagaraj, & Mann, 2014), as many proteins are not required for normal cellular function, but instead have important functions in specific circumstances. Dynamic regulation allows the proteome to be streamlined to optimize growth and survival. Consequently, numerous mechanisms exist to rapidly alter its composition during stress (Causton et al., 2001; Gasch et al., 2000; Simpson & Ashe, 2012).

### 1.2 Regulation of proteome composition at steady state

The four major processes involved in gene expression – transcription, mRNA decay, translation and protein decay – can all contribute both to determining ‘steady‐state’ protein levels and to dynamic changes in proteome composition. Despite an initial focus on transcriptional variation, the large impact of translational variation is now appreciated. Studies in mammalian cells at ‘steady‐state’ demonstrate a role for translation that is at least as great as that of transcription in determining protein levels globally (Schwanhäusser et al., 2011; Vogel et al., 2010). However, recent work suggests a more prominent role for transcription in yeast, with the majority of protein abundance variation explained by differences in mRNA abundance (Lahtvee et al., 2017). Furthermore, the contribution of each process to the expression of individual genes and sets of genes varies widely. For example, mRNA and protein levels correlate better for proteins with higher expression (Gygi, Rochon, Franza, & Aebersold, 1999). mRNAs whose abundance varies throughout the cell cycle show an especially strong correlation between mRNA and protein levels, in comparison with mRNAs that are constantly expressed (Greenbaum, Colangelo, Williams, & Gerstein, 2003). In contrast, such correlations are poorer for less well‐expressed proteins. This fits well with observations that the major translational control mechanisms involve repression of protein synthesis, as is outlined below.

### 1.3 Regulation of proteome composition during stress

Changing cellular conditions add further complexity to the control of gene expression, which does not necessarily reflect the importance of the different processes under ‘steady‐state’ conditions. Both transcriptional regulation and translational regulation make substantial, often complementary, contributions to altering the proteome during stress. The role of transcriptional regulation is illustrated clearly by the ‘environmental stress response’, in which shared, large‐scale changes in mRNA levels occur following a number of different stresses, including heat shock, oxidative stress and nitrogen starvation (Causton et al., 2001; Gasch et al., 2000). These stresses impose similar challenges, reflected in the functional themes shown in the up‐ and downregulated genes: translation, RNA metabolism and nucleotide biosynthesis functions are enriched among the downregulated genes, as cell growth is constrained (Gasch et al., 2000).

On the other hand, large‐scale translational changes also take place during stress. In many cases, significant repression of translation initiation can be observed within 1–30 min after the onset of stress (Ashe, De Long, & Sachs, 2000; Kershaw et al., 2015; Melamed, Pnueli, & Arava, 2008). Rapid translational inhibition provides a means for cells to bring about changes in the proteome without the time lag involved in transcription, mRNA processing, nuclear export and localization (Spriggs, Bushell, & Willis, 2010), and in some cases appears to act as a temporary response prior to longer‐term transcriptional reprogramming. Translational inhibition peaks after 60 min during high salt stress before recovering over the next 2 h, but transcriptional changes only first become apparent at this stage and peak later (Melamed et al., 2008). Likewise, global translation recovers slightly 3 h following glucose starvation (Vaidyanathan, Zinshteyn, Thompson, & Gilbert, 2014). In other cases the two responses are more closely coupled: the global extent of transcriptional and translational changes is similar within 30 min of exposure to hydrogen peroxide (Gerashchenko, Lobanov, & Gladyshev, 2012), although they differ after only 15 min, indicating that the translational response still precedes transcriptional changes (Costello et al., 2017). Furthermore, co‐directional regulation of the abundance and translation rate of many individual mRNAs occurs during stresses such as rapamycin treatment, heat shock (Preiss, Baron‐Benhamou, Ansorge, & Hentze, 2003), amino acid starvation (Halbeisen & Gerber, 2009; Smirnova et al., 2005) and osmotic shock (Halbeisen & Gerber, 2009). This phenomenon, termed ‘potentiation’ (Preiss et al., 2003), is believed to allow the amplification of changes in gene expression to generate a stronger and faster response. The effect appears reduced or absent during some other stress conditions, including butanol stress (Smirnova et al., 2005) and oxidative stress (Gerashchenko et al., 2012; Shenton et al., 2006), indicating its context dependence. Conflicting results have been reported for glucose starvation (Arribere, Doudna, & Gilbert, 2011; Castelli et al., 2011; Zid & O’Shea, 2014), suggesting that sampling time following stress influences the extent to which potentiation is observed under these conditions.

Taken together, these studies indicate that both transcriptional and translational regulation make substantial contributions to global stress‐induced changes in gene expression, although the precise effect of each and the balance between them vary widely between stresses. This review will focus on translational aspects of yeast stress responses, in particular on the protein factors that are involved in regulating translation initiation on specific sets of mRNAs.

## 2 GLOBAL MECHANISMS OF TRANSLATIONAL REPRESSION

### 2.1 Overview of the translation process

Translation is a hugely complex process requiring the coordinated action of many factors, and consequently offers many targets for regulation. It is classically subdivided into three major phases: initiation, elongation and termination. A detailed review of yeast translation was published recently (Dever, Kinzy, & Pavitt, 2016), so here only a very brief overview is given, with further details discussed in relation to the relevant regulatory mechanisms below.

**Figure 1:**
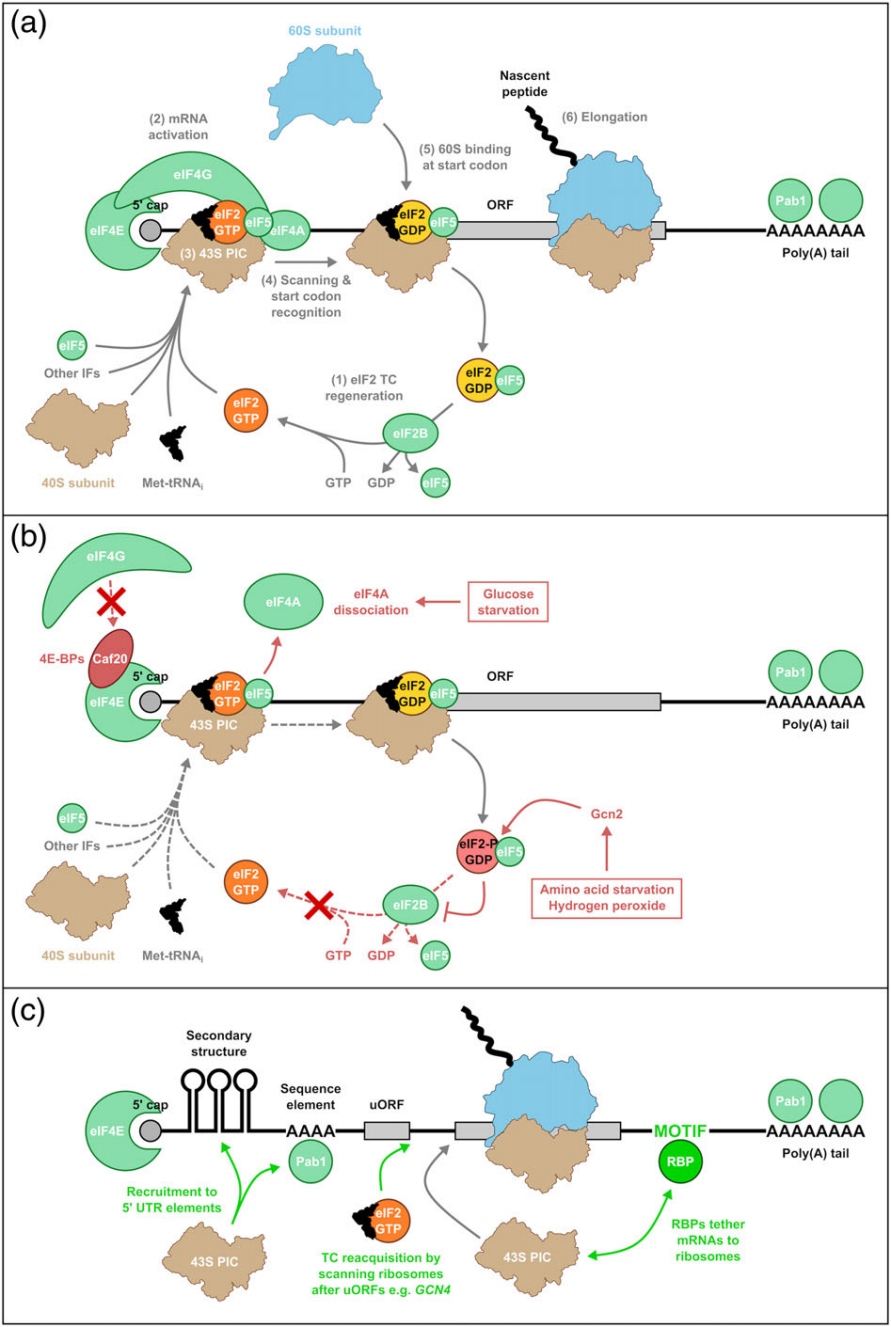
Global and specific regulation of translation initiation. (a) Overview of the major steps (numbered) in the cap‐dependent translation initiation pathway in Saccharomyces cerevisiae. (b) Global mechanisms for translational repression in response to different stresses. Mechanisms that interfere with the initiation pathway are shown in red. (c) Overview of mechanisms that promote translational upregulation or maintenance of specific mRNAs contrary to global stress‐induced repression (shown in green). Note that the elements and mechanisms shown may also combine to influence translation negatively, depending on their context [Colour figure can be viewed at wileyonlinelibrary.com]

In common with the mechanism in other eukaryotes, numerous initiation factors (IFs) recruit the small ribosomal subunit and charged methionyl‐initiator tRNA (Met‐tRNA_i_) to the 5′‐capped end of mRNAs (Figure 1a). This complex migrates in a 3′ direction to the start codon of the ORF (typically the first AUG encountered), where an interaction between the start codon and the tRNA anticodon is established. Recruitment and joining of the large ribosomal subunit readies the complex for peptide bond formation during elongation (Dever et al., 2016). Elongation factor 1A (eEF1A) delivers elongator tRNAs that decode the mRNA nucleotide sequence codon by codon. Ribosomes then translocate precisely along the ORF to the next codon, a reaction promoted by eukaryotic elongation factor 2 (eEF2). A fungal‐specific elongation factor, eEF3, facilitates the release of deacylated tRNAs from the ribosome. Once a stop codon is reached, release factors terminate the peptide chain and ribosomes and mRNA are recycled. It is thought that once a ribosome has transitioned from initiation to elongation and cleared the start codon, a new initiation event can commence so that multiple ribosomes translate a single mRNA simultaneously.

### 2.2 Phosphorylation of eIF2 and inhibition of eIF2B

A major aspect of the translational response to stress is the repression of protein synthesis globally, given the constraints on energy and nutrient availability (Spriggs et al., 2010). Initiation is the most complex phase of translation and believed to be rate‐limiting on many or most mRNAs, making it a good control point for many regulatory mechanisms to act (Spriggs et al., 2010). The global translational response to various stresses in yeast centres on the crucial process of eIF2‐GTP regeneration (Simpson & Ashe, 2012). Eukaryotic initiation factor 2 (eIF2) is responsible for delivering Met‐tRNA_i_ to the P site of the ribosome as part of a ternary complex (TC) with bound GTP (Dever et al., 2016). TC forms a 43S pre‐initiation complex (PIC) in concert with other initiation factors including eIF5 (Figure 1a). TC is critical for start codon recognition and hydrolysis of eIF2‐bound GTP, stimulated by eIF5, follows to trigger release of eIF2‐GDP/eIF5. In this latter complex eIF5 and eIF2*β* prevent GDP dissociation from eIF2*γ* (Jennings & Pavitt, 2010). eIF2 must be recycled to its GTP‐bound form by eIF2B, a factor that displaces eIF5 (Jennings, Zhou, Mohammad‐Qureshi, Bennett, & Pavitt, 2013) and performs guanine nucleotide exchange to promote eIF2‐GTP regeneration and TC formation before TC can bind eIF5 and form a new PIC to repeat the process of Met‐tRNA_i_ delivery (Jennings, Kershaw, Adomavicius, & Pavitt, 2017).

Depletion of charged aminoacyl‐tRNAs during stress activates the kinase Gcn2 (Hinnebusch, 2005), which phosphorylates eIF2*α* – its only known substrate (Dey et al., 2005) – on Ser51. Phosphorylation increases the affinity of eIF2 for eIF2B and transforms it from a substrate to a competitive inhibitor of the GEF (Jennings et al., 2017; Krishnamoorthy, Pavitt, Zhang, Dever, & Hinnebusch, 2001). The GTP‐bound form of eIF2 therefore cannot be regenerated so translational initiation is reduced for most mRNAs, given its central role in delivering Met‐tRNA_i_ to the ribosome on all cytoplasmic mRNAs (Figure 1b; Dever et al., 2016). Recently a second fail‐safe level of control has been identified, whereby eIF2B can bind to and inhibit phosphorylated TC and TC/eIF5 complexes, providing an alternative route to inactivate eIF2/eIF2B complexes (Jennings et al., 2017). It has also been established that the GEF activity of yeast eIF2B may be repressed independently of eIF2*α* phosphorylation. Translation initiation is rapidly repressed in response to excess fusel alcohols, which act as indicators of nitrogen scarcity, and eIF2B mutations alter this response (Ashe, Slaven, De Long, Ibrahimo, & Sachs, 2001; Taylor et al., 2010). However, eIF2B inhibition is not the only mechanism by which global translational activity can be repressed during stress.

### 2.3 RNA helicases: eIF4A and Ded1

Translational inhibition during glucose starvation is the most rapid of any acute stress, occurring in less than 1 min (Ashe et al., 2000). It is also independent of eIF2 phosphorylation (Ashe et al., 2000), although eIF2 phosphorylation increases at later times (Yang, Wek, & Wek, 2000), and instead rapid dissociation of the ATP‐dependent RNA helicase eIF4A from PICs is implicated as contributing to reducing initiation (Castelli et al., 2011). eIF4A is required to unwind secondary structure close to the 5′ cap to enable PIC binding. It further functions to unwind RNA secondary structure during scanning. The loss of helicase activity is therefore hypothesized to prevent PIC recruitment to mRNA 5′ ends or to lead to PIC stalling during scanning (Figure 1b; Castelli et al., 2011). It remains unclear how eIF4A is inhibited and released from PICs, and whether this mechanism is unique to glucose starvation. Ded1 is a second RNA helicase that has a role in PIC scanning and appears to be more important than eIF4A for unwinding secondary structures on many mRNAs, as many more structured mRNAs show altered translational efficiency in Ded1 conditional mutants (Sen, Zhou, Ingolia, & Hinnebusch, 2015). Ded1 may function independently or in concert with eIF4A and eIF4G (Gao et al., 2016). It is not clear how Ded1 activity is altered by cellular stress, but the polysome association of mRNAs with less structured leaders, such as those encoding components of the pentose phosphate pathway, is relatively resistant to glucose starvation (Castelli et al., 2011).

### 2.4 The closed‐loop complex and mRNA selection

Almost all eukaryotic mRNAs are thought to require the formation of a closed‐loop complex (CLC) for their efficient translation. The cap‐binding protein eIF4E and the poly (A) tail‐binding protein Pab1 recognize the 5′ and 3′ ends, respectively, of mRNAs. The 5′ cap and poly (A) tail act synergistically to promote translation: eIF4E and Pab1 are connected by the scaffold protein eIF4G to form the CLC (Hentze & Preiss, 1998). This mechanism is proposed to work either to promote efficient recycling of terminating ribosomes to initiate at the 5′ end of the same mRNA or as a quality control mechanism to ensure only intact mRNAs engage the translational machinery (Prévôt, Darlix, & Ohlmann, 2003). Furthermore, eIF4G helps to recruit the 43S PIC (the 40S ribosomal subunit and associated initiation factors) to the 5′ end of the mRNA to promote translation initiation (Figure 1a).

However, the components of this complex do not bind as uniformly to mRNAs as anticipated (Costello et al., 2015). While many mRNAs strongly associate with the CLC components, others appear much less dependent on these proteins for their translation. Indeed, some highly translated mRNAs are relatively depleted for them, suggesting the existence of alternative mechanisms to promote their translation (Costello et al., 2015). On the other hand, these data could indicate that there is a step in the standard initiation pathway where eIF4E and eIF4G are less stably bound to mRNAs, such as during 60S joining (Amrani, Ghosh, Mangus, & Jacobson, 2008; Wang et al., 2016). Recently it was shown that many mRNAs show similar reciprocal changes in translational efficiency and binding to both eIF4E and eIF4G during glucose starvation, amino acid starvation and oxidative stress (Costello et al., 2017). mRNAs that become more enriched in binding to the CLC components following stress have reduced translational efficiency, and vice‐versa. Together these data suggest that translationally repressed mRNAs may adopt a more stable interaction with the CLC factors, whereas actively translated mRNAs cycle through a step, possibly 60S joining, where CLC affinity for the mRNA is reduced.

The formation of the CLC can be regulated by the eIF4E‐binding proteins (4E‐BPs), which bind to eIF4E via an interaction motif matching that in eIF4G (Altmann, Schmitz, Berset, & Trachsel, 1997; Mader, Lee, Pause, & Sonenberg, 1995). The 4E‐BPs therefore block the eIF4E–eIF4G interaction and prevent CLC formation to antagonize PIC recruitment and repress 5′ cap‐dependent translation (Gingras, Raught, & Sonenberg, 1999). In mammalian cells the 4E‐BPs act downstream of mTOR signalling to repress translation in response to nutrient starvation (Richter & Sonenberg, 2005). S. cerevisiae contains two 4E‐BPs, Caf20 and Eap1, that share no sequence similarity with either each other or the mammalian 4E‐BPs outside the core eIF4E‐interaction motif (Gingras et al., 1999). They may be involved in translational regulation during stress, although the evidence in support of this is less extensive than for the mammalian proteins. Caf20 and Eap1 are both independently required for the induction of pseudohyphal growth during nitrogen starvation (Ibrahimo, Holmes, & Ashe, 2006; Park, Hur, Ka, & Kim, 2006). Furthermore, Eap1 mutants have altered responses to oxidative stress (Mascarenhas et al., 2008) and lipid stress (Deloche, de la Cruz, Kressler, Doère, & Linder, 2004).

Each of the yeast 4E‐BPs binds to around 1500 mRNAs under normal growth conditions, with over 1000 of these common to both (Costello et al., 2015). Caf20 target mRNAs typically have longer ORFs and are more poorly expressed than average (Castelli et al., 2015). However, around a quarter of these bind to Caf20 independently of eIF4E, instead interacting with the protein through elements in their 3′ untranslated regions (UTRs) (Castelli et al., 2015). The regulatory activity of Caf20 on these mRNAs is thought to involve direct interactions with ribosomes and potentially other regulatory proteins. Similarly, Eap1 is part of the multi‐protein SESA complex that forms on mRNAs such as *POM34*, which is involved in their specific translational repression (Sezen, Seedorf, & Schiebel, 2009). These studies together demonstrate the complexity of the regulatory role of the yeast 4E‐BPs, as it is now clear that they have the capacity to specifically regulate the translation of subsets of mRNAs, as well as to repress translation globally.

### 2.5 Elongation

Since it is a major rate‐limiting step of translation, the initiation phase is the predominant target for translational regulatory mechanisms. However, control may also be exerted at the elongation and termination phases of translation. As the phases of translation are linked, slow elongation rates at the start of the elongation phase can also reduce rates of initiation by altering the rate at which ribosomes clear the start codon (Chu et al., 2014). Furthermore, regulation of elongation occurs during stresses including amino acid starvation (Pelechano, Wei, & Steinmetz, 2015) and oxidative stress (Pelechano et al., 2015; Shenton et al., 2006). Under these conditions the accumulation of uncharged tRNAs can stall elongating ribosomes, in addition to their role in activating the eIF2 kinase Gcn2 (Zaborske et al., 2009). Extensive tRNA modification and binding of damaged tRNAs to ribosomes during oxidative, amino acid and cold‐shock stresses has recently been revealed, which probably contributes to both of these processes (Chen & Tanaka, 2018). Furthermore, ribosomes stall at His codons following the addition of 3‐aminotriazole, while stalling occurs instead at Asp and Ser codons during oxidative stress (Pelechano et al., 2015). Stress induced tRNA‐modification can also enhance the translation of some mRNAs with altered codon bias, including the ribosomal protein Rpl22a, but not its paralogue Rpl22b (Chan et al., 2012). It was found that the proportion of tRNA ^Leu(CAA)^ containing 5‐methylcytosine at the wobble position increases during oxidative stress, which enables selective translation of mRNAs enriched in UUG Leu codons (Chan et al., 2012). A number of other studies also provide evidence for a range of tRNA wobble modifications occurring during different stress conditions (Alings, Sarin, Fufezan, Drexler, & Leidel, 2015; Chan et al., 2010, 2015; Fernández‐Vázquez et al., 2013; Tigano et al., 2015). These probably contribute to biases in preferred codon usage and therefore contribute to the regulation of protein synthesis at the elongation phase during stress.

Another mechanism involved in translational regulation during stress is the phosphorylation of eEF2. In mammals, eEF2 phosphorylation impairs its interaction with ribosomes (Carlberg, Nilsson, & Nygard, 1990) and consequently inhibits ribosomal translocation (Spahn et al., 2004; Taylor et al., 2007). Likewise, yeast eEF2 is phosphorylated by the kinase Rck2 during osmotic stress (Teige, Scheikl, Reiser, Ruis, & Ammerer, 2001). Rck2 is essential for tolerance to osmotic stress, underlining the importance of elongation regulation under these conditions (Kumar, Hart, Wimalasena, Tucker, & Greetham, 2015). Furthermore, the master regulator Hog1, which activates Rck2, is also activated by independent mechanisms during both endoplasmic reticulum (ER) stress (Bicknell, Tourtellotte, & Niwa, 2010) and oxidative stress (Lee et al., 2017), implicating elongation control by eEF2 phosphorylation under these conditions. Together, these studies indicate that the simultaneous repression of multiple phases of translation contribute towards modulating protein synthesis during stress.

### 2.6 The fate of inhibited mRNA

Many mRNAs accumulate with some translation factors in cytoplasmic foci termed P‐bodies and stress granules following exposure to a range of stresses including glucose starvation, heat shock, sodium azide stress and hydrogen peroxide stress (Buchan & Parker, 2009; Buchan, Yoon, & Parker, 2011; Grousl et al., 2009; Hoyle, Castelli, Campbell, Holmes, & Ashe, 2007). Numerous RNA‐binding proteins co‐localize with these granules during glucose starvation (Mitchell, Jain, She, & Parker, 2013), while signalling complexes such as TORC1 also accumulate in stress granules during heat shock (Takahara & Maeda, 2012). There is substantial overlap in the protein and mRNA constituents of P bodies and stress granules, and their components are thought to undergo dynamic exchange (Buchan, Muhlrad, & Parker, 2008; Kedersha et al., 2005). However, the composition of these granules varies considerably between stress conditions (Buchan et al., 2011), which may reflect the differential translational needs of cells during different stresses. Likewise, there are distinct phases of mRNA recruitment to P bodies, with some mRNAs accumulating as early as 10 min following glucose deprivation, while other remain diffuse throughout the cytoplasm until much later (Simpson, Lui, Kershaw, Sims, & Ashe, 2014). P bodies are thought to be sites of mRNA storage and/or decay, owing to the co‐localization of multiple mRNA decay factors in these foci. On the other hand, stress granules probably represent a reservoir of inactive mRNAs, translation factors and associated proteins that can be re‐activated into the translating pool of mRNA following stress resolution, or that can be degraded by the mRNA‐decay machinery or the autophagy pathway during prolonged periods of stress (Buchan, Kolaitis, Taylor, & Parker, 2013).

## 3 mRNA‐SPECIFIC REGULATION: ROLES OF mRNA ELEMENTS

Global regulatory mechanisms allow the coordinated repression of many mRNAs, but these are only half of the story. It is hugely important for cell survival that certain groups of mRNAs are co‐ordinately upregulated contrary to global responses. For example, oxidoreductases, chaperones and antioxidants are all synthesized during oxidative stress (Shenton et al., 2006; Vogel, Silva, & Marcotte, 2011) in order to remove peroxide radicals and counter their damaging effects. Likewise, the response to amino acid starvation requires the upregulation of amino acid biosynthesis enzymes, along with amino acid permeases and protein degradation factors, which ‘scavenge’ to replenish the pool of intracellular amino acids (Halbeisen & Gerber, 2009; Smirnova et al., 2005). Countering global translational repression on specific sets of mRNAs is therefore crucial, and requires multiple elements which together bring about an appropriate translational response. RNA‐binding proteins (RBPs) and specialization of ribosomes can both contribute towards fine‐tuning protein expression to suit changing conditions (Figures 1c & 3b). However, regulation by either of these mechanisms would not be possible without *cis*‐acting elements within mRNAs to direct the interactions of PICs, RBPs and ribosomes towards specific mRNAs or groups of mRNAs.

### 3.1 Upstream ORFs

Sequence or structure elements within mRNAs can drastically alter their responses to translational regulatory mechanisms such as eIF2*α* phosphorylation, allowing ‘escape’ from global inhibition in an mRNA‐specific manner. One very well‐characterized mechanism, ribosome reinitiation, utilizes the presence of multiple short upstream ORFs (uORFs) in the 5′ UTRs of mRNAs. The *GCN4* mRNA contains four uORFs encoding di‐ or tripeptides (Hinnebusch, 1984), at which scanning ribosomes initiate before reaching the main ORF. Following translation of uORFs 1 and 2, the surrounding 5′ and 3′ sequences promote the retention of 40S–mRNA interactions, including via interactions with eIF3, and thereby promote the resumption of scanning downstream (Grant & Hinnebusch, 1994; Grant, Miller, & Hinnebusch, 1995; Gunišová, Beznosková, Mohammad, Vlčková, & Valášek, 2016; Mohammad, Pondelícková, Zeman, Gunisová, & Valásek, 2017; Munzarová et al., 2011; Szamecz et al., 2008). These 40S ribosomes are able to reinitiate should they reacquire Met‐tRNA_i_ as part of TC. High eIF2B activity under normal conditions leads to rapid reacquisition of TC and translation of uORF 3 or 4. These uORFs are not competent for reinitiation and consequently promote ribosome dissociation so that low levels of *GCN4* translation are maintained during non‐starvation conditions (Miller & Hinnebusch, 1989). Conversely, reduced eIF2B GDP/GTP exchange activity during stress allows ribosomes to migrate past uORFs 3 and 4 before they reacquire TC. Therefore, ribosomes that then bind TC before reaching the *GCN4* start codon will reinitiate at the main ORF (Abastado, Miller, Jackson, & Hinnebusch, 1991). As a result, Gcn4 protein is synthesized in a ‘paradoxical’ manner during stress so that it can upregulate transcription of stress‐responsive genes. Gcn4 activates transcription of amino acid biosynthetic enzymes, so its low expression during normal conditions and specific induction during starvation ensures tight control of cellular resources.

Another well‐studied system that exemplifies uORF‐mediated regulation is the *CPA1* mRNA, which contains a single uORF encoding the arginine attenuator peptide (AAP) (Werner, Feller, Messenguy, & Piérard, 1987). Cpa1 catalyses a step in arginine biosynthesis so is only needed when arginine is scarce. In this case, leaky scanning of the *AAP* uORF leads to AAP translation by only ~50% of ribosomes, so that 40S ribosomes that migrate past the uORF can translate the main ORF to produce Cpa1. However, when arginine is present ribosomes become stalled during translation of AAP and prevent any ribosomes from reaching the main ORF, thus reducing Cpa1 synthesis. Ribosome stalling requires an interaction between arginine and the AAP peptide within the ribosome exit channel, providing highly specific regulation of *CPA1* translation (Gaba, Wang, Krishnamoorthy, Hinnebusch, & Sachs, 2001; Wang, Gaba, & Sachs, 1999).

*GCN4* and *CPA1* exemplify two distinct mechanisms by which uORFs can regulate translation. Although they are relatively rare, being found in only 13% of yeast mRNAs (Lawless et al., 2009), many other uORFs have important effects on the translation of their associated ORFs. Ribosome footprinting has shown that many uORFs are differentially translated during stress (Ingolia, Ghaemmaghami, Newman, & Weissman, 2009), while studies on individual uORF‐containing mRNAs provide further evidence for regulatory roles over a range of conditions (Blank et al., 2017; Zhang & Dietrich, 2005).

### 3.2 Alternative start codons

Leaky scanning also occurs on non‐uORF containing mRNAs and can act as a mechanism for alternative start codon selection, whereby a portion of ribosomes initiate at a downstream, in‐frame start codon. Most ORFs initiate with AUG start codons, although there is increasing evidence of initiation at other start codons, albeit often at a reduced frequency (Kearse & Wilusz, 2017). Several *N*‐terminally extended proteins have been observed by ribosome footprinting following oxidative stress (Gerashchenko et al., 2012). In some cases, the longer form of the protein includes a signal sequence that is absent from the shorter form, localizing the two forms to different subcellular compartments. For example, Gpx3 and Grs1 are both localized to mitochondria via the addition of a targeting sequence produced from an upstream non‐AUG start codon (Chang & Wang, 2004; Kritsiligkou et al., 2017), whereas their shorter forms are found in the cytoplasm. The longer, mitochondrial form of Gpx3 is observed following the addition of hydrogen peroxide, suggesting that stress‐induced regulatory mechanisms influence non‐AUG‐initiated translation on its mRNA.

### 3.3 Other sequence elements

Specific sequence elements in mRNAs can recruit *trans*‐acting factors that can confer differential sensitivity to translational regulatory mechanisms to tailor global responses. Initiation on specific yeast invasive growth mRNAs in response to starvation is dependent on the binding of Pab1 to unstructured A‐rich sequences in their long 5′ UTRs (Figure 1c; Gilbert, Zhou, Butler, & Doudna, 2007). Pab1, which normally binds to poly (A) tails, is proposed to assist in cap‐independent recruitment of eIF4G and PICs on such mRNAs to drive their translation and consequently bring about the morphological changes required following stresses such as glucose starvation (Gilbert et al., 2007). Likewise, some A‐rich 5′ UTRs were found to specifically enhance translation during amino acid starvation, suggesting that they may work by a similar mechanism (Rachfall, Heinemeyer, Morgenstern, Valerius, & Braus, 2011). On the other hand, U‐rich 5′ UTRs are preferentially bound by eIF4G in unstressed cells, which may promote the efficient translation of mRNAs containing these elements (Zinshteyn, Rojas‐Duran, & Gilbert, 2017).

### 3.4 Secondary structure elements

Yeast 5′ UTRs are typically unstructured, but some contain stem‐loop structures (Kertesz et al., 2010). Efficient scanning, and therefore translation, of such mRNAs relies upon the action of the RNA helicases eIF4A and Ded1 (Sen et al., 2015). Secondary structure elements can affect the degree to which specific mRNAs respond to some global translational regulatory mechanisms. Indeed, the attenuation of polysome association during glucose starvation is more severe on mRNAs with highly structured 5′ UTRs, as these mRNAs rely more heavily on eIF4A and Ded1 activity (Castelli et al., 2011).

Internal ribosome entry sites (IRESs) are elements that allow translation of mRNAs in a cap‐independent manner (Jackson, 2013). They are typically structured and in mammalian cells represent a strategy for viral mRNAs to prioritize their own translation while the cap‐dependent translation of host mRNAs is repressed. Up to 10% of mammalian cellular mRNAs are estimated to contain IRES sequences, where they serve a similar function to promote translation during stress (Mitchell et al., 2005; Sarnow, 1989). However, their prominence in yeast is less clear, as is their mechanism of translation initiation. The 5′ UTR of *URE2* contains a proposed IRES that is required for synthesis of the shorter functional form of the protein (Komar et al., 2003). Translation initiation at this element is repressed by the alternative tRNA‐binding factor eIF2A (Komar et al., 2005). This protein is unstable, with a half‐life of only 17 min (Komar et al., 2005), and it is proposed that translation from the *URE2* IRES element may increase during stress if the turnover of the protein is increased (Reineke & Merrick, 2009). However, the extent of eIF2A involvement in yeast stress responses, as well as its mechanism of action, is not yet clear.

Secondary structure elements may also act as binding sites for other regulatory proteins. For example, the *ASH1* mRNA contains stem loops in both its ORF and 3′ UTR, which mediate binding to the RBPs Khd1 and Puf6, respectively. Both of these RBPs repress *ASH1* translation by different mechanisms: Khd1 interacts with eIF4G to form an inhibitory CLC, whereas Puf6 is proposed to prevent 60S joining and initiation mediated by eIF5B (Figure 1c). Spatially regulated phosphorylation of both RBPs relieves their repressive effects upon localization to the bud tip (Deng, Singer, & Gu, 2008; Paquin et al., 2007), restricting synthesis of Ash1 to the appropriate location.

### 3.5 mRNA modifications

As indicated previously, all nuclear‐encoded mRNAs are modified by the addition of both a 5′ cap and 3′ poly (A) tail, which act synergistically to promote translation (Tarun & Sachs, 1995) via recruitment of the CLC. Both of these modifications are removed during mRNA decay, and hence variations in poly (A) tail length can contribute to the control of translation in many organisms, including yeast (Beilharz & Preiss, 2007; Park, Yi, Kim, Chang, & Kim, 2016; Weill, Belloc, Bava, & Méndez, 2012). Deadenylation is inhibited by heat shock, hyperosmolarity and glucose deprivation, suggesting that variable polyadenylation could play a part in the cellular response to various stresses, although the role of this in regulating translation under these conditions is not yet clear (Hilgers, Teixeira, & Parker, 2006).

More recently, other modifications including the methylation of internal adenosine nucleotides to form N^6^‐methyladenosine (m^6^A) have emerged as means to regulate mRNA functions such as protein synthesis in a variety of contexts, including in response to stress (Roundtree, Evans, Pan, & He, 2017; Zhou et al., 2018). In yeast, although m^6^A is widespread across different mRNAs, its presence is restricted to meiosis (sporulation), where m^6^A patterns vary over time (Schwartz et al., 2013). The developmental process of sporulation occurs in response to nutrient scarcity and involves extensive translational control, as evident from ribosome profiling experiments (Brar et al., 2012). While the precise roles of m^6^A during meiosis are not yet clear, m^6^A‐containing mRNAs are enriched on polysomes, suggesting that they are preferentially translated over non‐m^6^A‐containing mRNAs (Bodi, Bottley, Archer, May, & Fray, 2015).

In summary, mRNAs therefore contain the regulatory elements, both sequence‐ and structure‐based, that regulate their translational fate by modulating interactions with both translation initiation factors and ribosomes (Figure 1c). As indicated above, interactions with other *trans*‐acting protein factors are required to transduce these signals into effects on protein expression. Selected RBPs that have roles in this process are discussed in the next section.

## 4 mRNA‐SPECIFIC REGULATION: ROLES OF mRNA‐BINDING PROTEINS

A rich network of mRNA–protein interactions (Hogan, Riordan, Gerber, Herschlag, & Brown, 2008) regulates the fate of mRNAs at multiple stages of their life, determining their splicing, nuclear export, localization, translation and degradation (Moore, 2005). RNA‐binding proteins are thought to contribute to the formation of ‘post‐transcriptional operons’ which regulate the fate of hundreds or thousands of mRNA targets, many of which may be functionally related (Keene, 2007). Through binding to sequence motifs or secondary structure elements (Freeberg et al., 2013) shared across large sets of mRNAs, RBPs can simultaneously control mRNA localization, translation and degradation to bring about coordinated functional effects. As a result, they can play important roles in the rapid post‐transcriptional reprogramming of gene expression following changes in external conditions.

**Figure 2:**
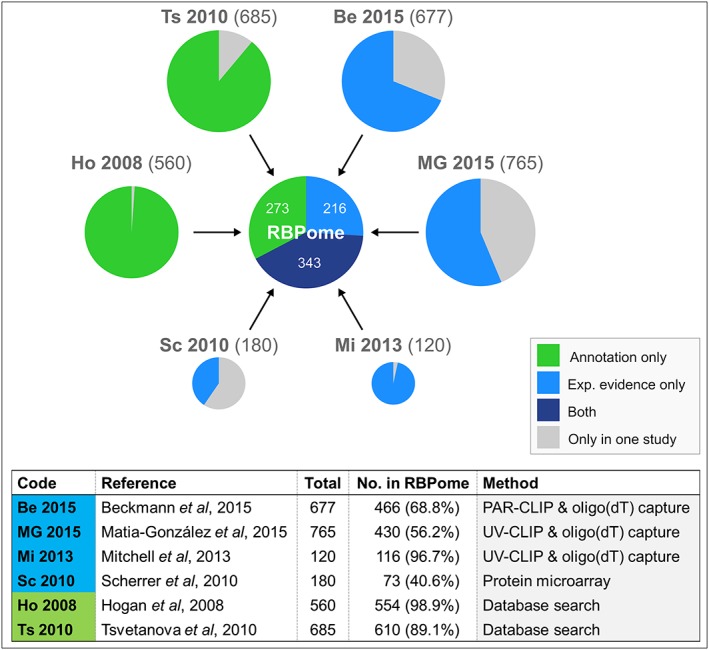
The S. cerevisiae RBPome consists of over 800 proteins that reproducibly associate with mRNA. The yeast RBPome was constructed by integrating evidence from six independent studies that conducted proteome‐wide surveys of mRNA binding (references in the table). A protein was considered part of the RBPome if it was determined to bind mRNA in two or more of these studies. The proportion of the RBPs from each study that are present in the RBPome is indicated, as is the proportion with annotation evidence only, experimental evidence or both [Colour figure can be viewed at wileyonlinelibrary.com]

Taking together sequence‐based predications and experimental evidence, over 800 S. cerevisiae proteins make up the yeast ‘RNA‐binding proteome’ (RBPome; Figure 2; Beckmann et al., 2015; Hogan et al., 2008; Matia‐González, Laing, & Gerber, 2015; Mitchell et al., 2013; Scherrer, Mittal, Janga, Gerber, & Botstein, 2010; Tsvetanova, Klass, Salzman, & Brown, 2010). Many of these contain classically recognized RNA‐binding motifs or domains such as the RNA recognition motif (RRM), heterogenous nuclear ribonucleoprotein K homology (KH) domain, La motif (LaM) and Pumilio family (PUF) domain (Lunde, Moore, & Varani, 2007). However, a substantial proportion of the RBPome lacks these classical domains and there is now substantial evidence for non‐canonical ‘moonlighting’ roles of, for example, metabolic enzymes as RNA binders (Castello et al., 2012; Castello, Hentze, & Preiss, 2015; Hentze & Preiss, 2010). New RBPs continue to be discovered, while the ‘classical’ functions of many proteins are now being reassessed (Hentze, Castello, Schwarzl, & Preiss, 2018). It is clear that much of the complex network of RNA–protein interactions, and importantly its roles in modulating protein expression, still remains to be uncovered. However, there are numerous clear examples where mRNA interactions with protein binding partners play significant roles (both directly and indirectly) in translational regulation during changing cellular conditions. A selection of protein players involved in such regulatory processes is discussed below.

### 4.1 Pumilio family proteins

The Pumilio family (Puf) proteins have a well‐documented role in mRNA binding, RNA decay and translational control. The six different family members in yeast all bind distinct sets of mRNAs with shared functions (Gerber, Herschlag, & Brown, 2004) to regulate their fate. For example, a major class of Puf3 target mRNAs is localized to the mitochondrial periphery (Saint‐Georges et al., 2008) and has functions relating to mitochondrial biogenesis and respiration, while Puf4 associates predominantly with mRNAs encoding nucleolar proteins, a role it shares with Puf5 (Gerber et al., 2004). Closely related motifs, typically within 3′ UTRs, direct binding to each of the proteins in the family (Gerber et al., 2004). Furthermore, Puf3, Puf4 and Puf5 were recently shown to have an expanded and overlapping ‘super‐network’ of mRNA targets, with shared targets enriched in translation‐related functions (Lapointe et al., 2017). A role for Puf3 in translational regulation is inferred as target mRNAs show increased association with polysomes in its absence (Kershaw et al., 2015). A major function of Puf3 may be to spatially repress the translation of nuclear‐encoded mitochondrial mRNAs until they are at the mitochondrial periphery.

Furthermore, several Puf proteins have been implicated in post‐transcriptional responses to stress. Puf1 and Puf2 appear to play a role in the response to high calcium, possibly via regulation of *ZEO1* expression (Haramati et al., 2017). mRNAs bearing a Puf3‐binding motif are associated with translational downregulation following exposure to hydrogen peroxide, while the protein's polysome association also reduces under these conditions (Rowe et al., 2014). Puf3 becomes phosphorylated in its *N*‐terminal unstructured region during glucose starvation, which switches the fate of its target mRNAs by promoting their enhanced translation (Lee & Tu, 2015). This is consistent with Puf3 contributing to the cellular response to stress by promoting the expression of mitochondrially destined proteins for enhanced respiratory growth. Together, the Puf family proteins exemplify a class of RBPs that can co‐ordinately and bi‐directionally regulate the post‐transcriptional fate of specific sets of mRNA targets under a variety of cellular conditions.

### 4.2 La‐related proteins

While Puf3 appears to act predominantly as a translational repressor, it is important to switch on the expression of specific sets of mRNAs during stress. One way of rapidly achieving this is through the action of RBPs that act as translational activators to maintain or promote the translation of target mRNAs under such conditions.

The La family proteins, including yeast Lhp1, contain RRM domains adjacent to a family‐specific LaM to mediate RNA binding. Lhp1 functions as a chaperone for RNA polymerase III transcripts and has a role in their 3′ end maturation (Maraia, Mattijssen, Cruz‐Gallardo, & Conte, 2017). In contrast, the yeast La‐related proteins (LARPs), Slf1 and Sro9, possess the LaM but lack the adjacent RRM (Sobel & Wolin, 1999) and function in RNA metabolism predominantly in the cytoplasm. Both interact with translating ribosomes (Sobel & Wolin, 1999) and >500 mRNA targets, mostly shared between the two LARPs, have been identified (Kershaw, Costello, Castelli, et al., 2015; Schenk, Meinel, Strässer, & Gerber, 2012). Those mRNAs bound by Slf1 tend to be more abundant and highly translated, and include many that are involved in the copper ion and oxidative stress responses (Kershaw, Costello, Castelli, et al., 2015; Schenk et al., 2012). Slf1 appears to play a substantial role in translational response under these conditions: ~40% of proteins found to be upregulated following hydrogen peroxide treatment are encoded by Slf1 targets (Kershaw, Costello, Castelli, et al., 2015). Conversely, both yeast LARPs move from the cytoplasm to P bodies (Mitchell et al., 2013) and become hyperphosphorylated, along with a number of other RBPs, during glucose starvation (Chang & Huh, 2018).

**Figure 3:**
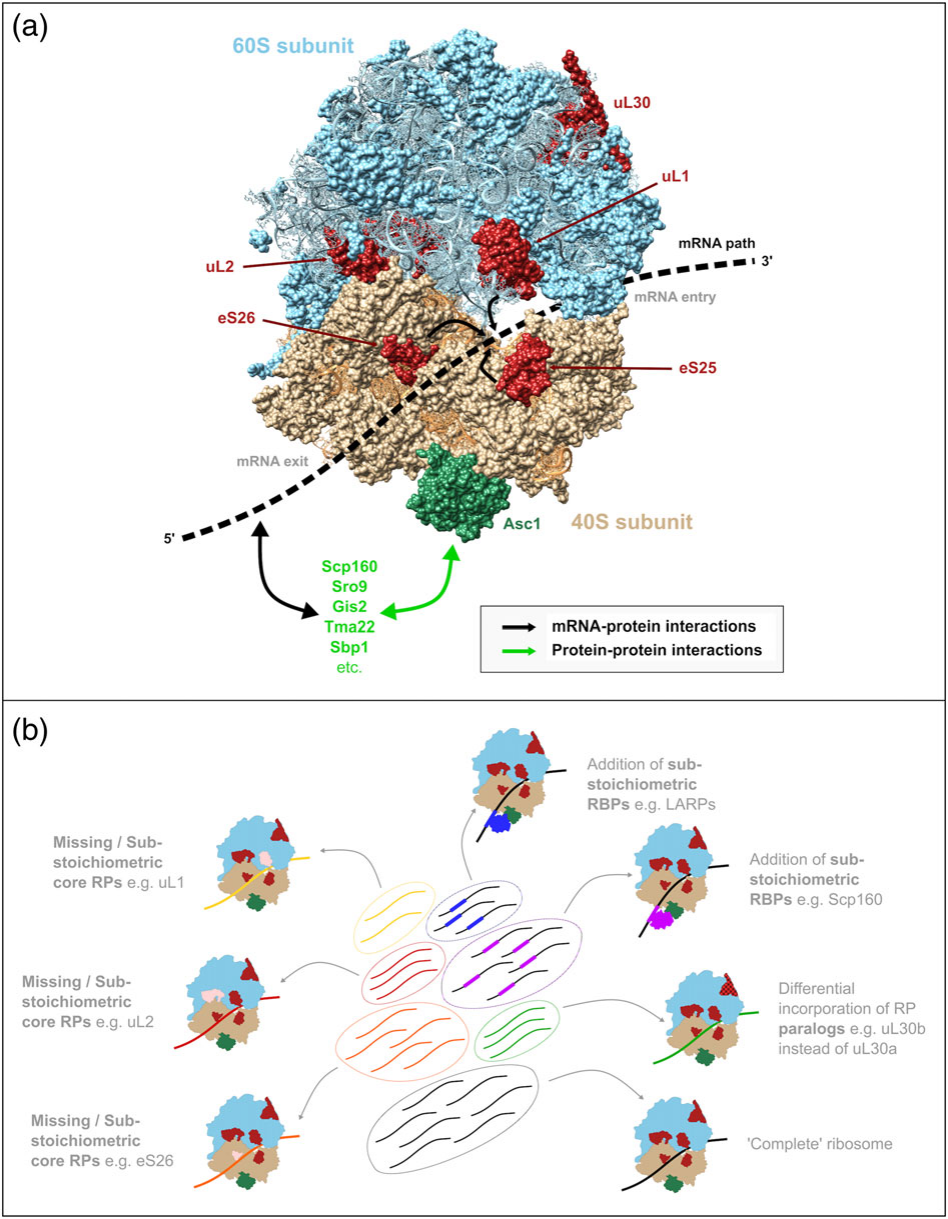
The role of ribosomal heterogeneity in translational regulation. (a) Surface representation of the yeast 80S ribosome, shown from the mRNA exit channel. Some ribosomal proteins that are discussed in the text are highlighted in dark red and dark green. RBPs that interact with Asc1 to tether specific mRNAs to ribosomes and specifically regulate their translation are shown in green text. 40S and 60S ribosome subunit structures were taken from Protein Data Bank files 4 V88 and 4V7R (Ben‐Shem et al., 2011), respectively, and drawn with UCSF Chimera software. (b) Illustration of the ribosome filter hypothesis (Mauro & Edelman, 2002, 2007). mRNAs do not interact uniformly with all ribosomes, and instead subsets of mRNAs (within ovals) interact with and are translated specifically by specialized sub‐sets of ribosomes. Cartoon ribosomes are shown in the same representation as in (a), with differential shading indicating a range of ways in which ribosomes can be specialized [Colour figure can be viewed at wileyonlinelibrary.com]

The strong polysome association of Slf1 and Sro9 under normal growth conditions (Sobel & Wolin, 1999) and during oxidative stress, combined with the upregulation of the proteins encoded by their target mRNAs (Kershaw, Costello, Castelli, et al., 2015), supports a model of the LARPs functioning as activators (or maintainers) of translation of specific sets of mRNAs during some adverse conditions. They may carry out this function by binding to both mRNAs and ribosomes to tether transcripts and thereby promote their translation. Indeed, both Sro9 and the human LARP4B bind to the 40S ribosomal protein Asc1/RACK1 (Figure 3a) (Opitz et al., 2017; Schäffler et al., 2010). How the yeast LARPs can activate the translation of these tethered mRNAs is not yet resolved.

### 4.3 Scp160

The yeast homologue of human vigilin, Scp160, is a highly conserved RBP containing 14 RNA‐binding KH domains, which also illustrates strong links between RNA binding and translational control. Scp160 associates with both cytoplasmic and ER‐associated polysomes (Frey, Pool, & Seedorf, 2001), for which its KH domains are required but not sufficient (Li et al., 2004). It binds to over 1000 mRNAs, including those involved in cell wall, plasma membrane, ER and nucleolus‐related functions (Hogan et al., 2008; Li, Watson, & Fridovich‐Keil, 2003), although the links between these and the biological functions it is implicated in remain unclear. Some mRNAs show an altered ‘translational state’ (i.e. a change in ribosome association) following depletion of Scp160 (Hirschmann et al., 2014), implicating it in the regulation of their translation. As Scp160 targets are enriched in the set of mRNAs showing high polysome association during glucose starvation, it may promote translation under these conditions (Arribere et al., 2011). Furthermore, *scp160∆* cells also show aberrant P‐body formation under non‐stress conditions (Weidner, Wang, Prescianotto‐Baschong, Estrada, & Spang, 2014), suggesting that it has a role in preventing their formation. In particular, the protein may enhance translation elongation, as the abundance of a target‐encoded protein (Pry3) was reduced in Scp160‐depleted cells, despite the mRNA increasing in polysome association (Hirschmann et al., 2014). To account for these and other observartions, Scp160 has been proposed to enhance translation elongation efficiency via promoting the re‐use of tRNAs locally (Hirschmann et al., 2014).

Studies of the physical interactions that Scp160 makes contribute further to this model. Protein–protein crosslinking identified Scp160 binding, via its C‐terminus, to both eIF1A and the 40S ribosomal subunit protein Asc1 (Figure 3a; Baum, Bittins, Frey, & Seedorf, 2004). This ribosomal contact was also observed much more recently using BioID to systematically identify Asc1‐proximal proteins (Opitz et al., 2017). As may be the case with the LARPs and some other RBPs, binding to ribosomes appears to allow Scp160 to tether its target mRNAs to the translational machinery and hence promote their translation. Acting as ‘middlemen’ between mRNAs and ribosomes is one mechanism by which it and other RBPs may influence mRNA fate to regulate gene expression. Furthermore, the sub‐stoichiometric binding of a variety of different RBPs to ribosomes can be viewed as a means of generating specialized ribosomes that are competent for the translation of specific subsets of mRNAs, favouring their translation above that of others to streamline protein production.

## 5 mRNA‐SPECIFIC REGULATION: RIBOSOMAL SPECIALIZATION AND TRANSLATIONAL CONTROL

It is now becoming clear that ribosomes are not all the same, with a rapidly growing body of evidence showing structural and functional differences between ribosomes isolated from the same cell population. Rather than containing uniform populations of ribosomes, it appears that cells contain multiple populations of ribosomes with diverse compositions, which each favour the translation of certain subsets of the transcriptome, as was suggested over 15 years ago as the ‘ribosome filter hypothesis’ (Figure 3b; Mauro & Edelman, 2002, 2007).

### 5.1 Sub‐stoichiometric ribosome‐associated proteins

Perhaps the most straightforward form that this can take is through the aforementioned association of sub‐stoichiometric RBPs to promote interactions with specific mRNAs. In addition to the RBPs described above there may be many others that interact with ribosomes and act in such a way: indeed, numerous proteins associate with polysomal and monosomal complexes (Fleischer, Weaver, McAfee, Jennings, & Link, 2006), but the functions of many of these remain unclear. Of particular importance for ribosomal interactions with RBPs is the 40S subunit protein Asc1 (Figure 3a), which was itself initially thought to be more peripherally associated than the other core ribosomal proteins. This protein contains a WD40 domain, which is made up of seven WD repeats (each around 40 amino acids) that together form a *β*‐propeller structure (Ben‐Shem et al., 2011; Ben‐Shem, Jenner, Yusupova, & Yusupov, 2010). The WD40 domain is widespread throughout eukaryotes and well‐known to support numerous protein–protein interactions, giving WD40‐containing proteins scaffolding roles in many cases. Through this domain, Asc1 interacts with canonical eIFs such as eIF3, contributing to the formation of pre‐initiation complexes (Des Georges et al., 2015; Kouba, Rutkai, Karásková, & Valášek, 2012). It also binds to a variety of other proteins including Scp160 (Baum et al., 2004; Opitz et al., 2017), Sro9 (Opitz et al., 2017), Tma22 (Fleischer et al., 2006; Gavin et al., 2002), the polysome and mRNA‐associated translational activator Gis2 (Opitz et al., 2017), the eIF4G‐interacting translational repressor Sbp1 (Gavin et al., 2006) and many more (Figure 3a). Each of these RBPs could prime ribosomes to translate a specific set of mRNAs, generating diversity in ribosomal composition and function. In addition, under nutrient‐rich conditions Asc1 is important for the optimal translation of highly translated mRNAs with short ORFs (Thompson, Rojas‐Duran, Gangaramani, & Gilbert, 2016). However, it is not yet clear how interactions with RBPs such as those discussed above modifies the translation of Asc1 target mRNAs, or how this is affected by changing environmental conditions.

### 5.2 Heterogeneous composition of ribosomes

Ribosomes are highly complex macromolecular structures comprisinf two subunits: the large/60S subunit, which in yeast contains the 26S, 5.8S and 5S rRNA and 46 ribosomal proteins (RPs), and the small/40S subunit, which contains the 18S rRNA and 33 RPs. The large subunit contains the peptidyl transferase centre, where peptide bonds are formed between the nascent polypeptide chain in the peptidyl site and the new amino acid in the acceptor site, as well as the polypeptide exit channel. The small subunit contains the decoding centre where tRNAs base‐pair to and decode the mRNA. The majority of the yeast RPs are encoded by two paralogous genes (Planta & Mager, 1998), enabling ribosomes to vary in their core subunit composition as well as in the association of peripheral RBPs. Many differences in the expression, localization and function of these paralogous RPs have been documented over the past 25 years (Carroll & Wickner, 1995; Dresios, Derkatch, Liebman, & Synetos, 2000; Kim, Ha, & Huh, 2009; Komili, Farny, Roth, & Silver, 2007; Ohtake & Wickner, 1995; Palumbo, Fuchs, Lutz, & Curcio, 2016; Petibon, Parenteau, Catala, & Elela, 2016), but direct evidence of structural differences between ribosomes has only begun to emerge more recently, with the rapid improvement in mass spectrometry and the analysis of the resulting data. A number of recent studies have probed stoichiometric differences between eukaryotic RPs (Shi et al., 2017; Slavov, Semrau, Airoldi, Budnik, & van Oudenaarden, 2015), in contrast to earlier structural work that portrayed ribosomes as having a uniform conformation (Ben‐Shem et al., 2010, 2011). An unfathomable variety of ribosomes could in theory be generated via differences in both composition and modification status of RPs and rRNAs (Dinman, 2016): paralogues of many of the core RPs can be differentially incorporated, rRNAs and RPs can be modified via, for example, the addition of methyl or phosphoryl groups, and some RPs may show differences in stoichiometry. However, it is likely that only a small proportion of these theoretical ribosome variants actually exist within cells, and many of these may not be functionally distinguished from others (Dinman, 2016). Nevertheless, differences are becoming apparent and at least some of variants appear to respond to different environmental stresses.

### 5.3 Paralogue‐specific differences in RP function

It is well documented that the yeast genome was duplicated over 2 million years ago, and many of the duplicated genes have been lost or modified substantially since. However, ribosomal proteins are highly prominent among the paralogue pairs that remain: 59 of the 80 RPs are present in ‘a’ and ‘b’ forms expressed from paralogous genes (Planta & Mager, 1998). Despite the fact that many of these protein pairs differ in only a few amino acids, the maintenance of both copies for so many of the RPs is suggestive of some differential function. Most of the RP paralogue pairs are expressed at different levels (Ghaemmaghami et al., 2003; Kulak et al., 2014), and genetic and biochemical studies have identified non‐equivalence in the behaviour of various of these, which may be related to different translational activity on mRNA substrates, among other effects (Segev & Gerst, 2018).

The RPs uL30a and uL30b [Rpl7a and Rpl7b in the former yeast naming system (Ban et al., 2014); Figure 3a] are 244 amino acid proteins which differ in only five of those. Despite this small structural difference, the pair appear to differ in their physiological roles in yeast. uL30a was among 12 paralogous RP genes identified in a genetic screen as having a random budding pattern, as well as a reduced growth rate, although uL30b was not (Ni & Snyder, 2001), and later work showed further phenotypic differences between the two deletion mutants, for example altered cell size, altered *ASH1* mRNA localization, and differences in sensitivity to various drugs and stressors (Komili et al., 2007). Furthermore, the localization of the uL30 paralogues differs, with the ‘b’ form found in both nucleus and cytoplasm, while the ‘a’ form is exclusively cytoplasmic (Kim et al., 2009). This difference does not result from the sequence divergence (Kim et al., 2009), so may be due to changes in non‐coding regions or expression of the respective mRNAs. For example, the uL30 paralogues each contain introns encoding different small nucleolar RNAs that both guide methylation of 25S rRNA at A807 (Samarsky & Fournier, 1999). A more recent study provided further evidence that differences in the loci, rather than in the proteins themselves, contribute to the paralogue‐specific functions for this pair (Palumbo et al., 2016), suggesting that, although the proteins have different physiological roles, their actual structure and function within ribosomes may be equivalent.

The RP paralogue pair uL1a and uL1b (Rpl1a and Rpl1b) are absolutely identical, but individual deletion mutants of the two also exhibit phenotypic differences. uL1b, but not uL1a, mutant strains are respiratory deficient, showing slow growth on fermentable carbon sources and none on non‐fermentable carbon sources (Segev & Gerst, 2018). Notably, two other RPs with highly similar paralogues, uL2b and eS26b (Rpl2b and Rps26b), show the same phenotype (Segev & Gerst, 2018). The uL1 mutant strains translate different mRNAs, as shown by PUNCH‐P (Aviner, Geiger, & Elroy‐Stein, 2013), with the uL1b deletion strain failing to efficiently translate mitochondrial and cell wall‐related proteins (Segev & Gerst, 2018). It remains unclear how uL1a‐ and uL1b‐containing ribosomes could differ in their translational activity, but it is again possible that non‐coding sequences could have an influence, perhaps via directing differential processing of the two mRNAs.

Altogether, the available evidence suggests that some RP paralogue pairs can exhibit marked differences in their physiological functions and impact the translation of specific groups of mRNAs, despite very similar amino acid compositions. Rather than randomly incorporating the ‘a’ or ‘b’ forms of RP paralogue pairs, preferentially generating ribosomes with one form or the other under stress conditions could act as a mechanism for specializing translation and responding to stress (Figure 3b). Recently a switch in the relative levels of the highly similar paralogue pair eL8a and eL8b (Rpl8a and Rpl8b) in 80S ribosomes was observed following a change in carbon source from glucose to glycerol (Sun et al., 2018). Genetic analysis provides further evidence for divergent functions of this paralogue pair, as the two can complement each other during growth on glucose but not glycerol (Sun et al., 2018). In this manner, the eL8a–eL8b switch illustrates one way in which dynamic ribosomal specialization may contribute to streamlining translation during stress.

### 5.4 Differential RP stoichiometry

In addition to such differences between paralogous RPs, other recent work suggests that some RPs may be present in ribosome populations at sub‐stoichiometric levels, which may have important consequences for ribosomal function. Such findings suggest that some RPs are dispensable for normal ribosomal function, and that their loss may even specialize ribosomes towards translation of certain mRNA substrates. The generation of heterogeneous ribosomes via subtraction of subunits (Briggs & Dinman, 2017) contrasts with the addition of RBPs to achieve a similar goal, but also appears to be important for translational regulation during changing conditions.

Mass spectrometry used in combination with polysome profiling (Slayter, Warner, Rich, & Hall, 1963; Warner & Knopf, 2002; Warner, Knopf, & Rich, 1963) shows that relative RP levels differ between polysomal and monosomal ribosomes in both yeast and mammals (Slavov et al., 2015). Furthermore, these ratios also vary under different growth conditions: some RPs have different profiles in yeast grown in ethanol compared with cells grown in glucose (Slavov et al., 2015). Related work in mammalian cells demonstrated sub‐stoichiometric association of uL1, eL38, eS7 and eS25 with heavily translated mRNAs in polysomes (Shi et al., 2017). Some of these RPs associate with certain subsets of mRNAs: transcripts with extracellular matrix organization, alcohol metabolism and other functions are enriched in association with uL1, whereas those with cell cycle, vesicle‐mediated transport and organelle fission functions are more highly associated with eS25 (Shi et al., 2017). Direct interactions of these RPs with mRNAs may be responsible for such effects, as both uL1 (Shi et al., 2017) and eS25 (Landry, Hertz, & Thompson, 2009) bind and are required for IRES‐mediated translation. In this manner, the sub‐populations of ribosomes containing these RPs can promote the translation of specific mRNAs, and it is likely that similar mechanisms exist in yeast.

While some core RPs show sub‐stoichiometric ribosome association during unstressed conditions, others appear to be regulated in a dynamic manner. eS26 (Rps26) is depleted from yeast ribosomes under high‐pH and high‐salt conditions, but notably not in the presence of another stressor, caffeine (Ferretti, Ghalei, Ward, Potts, & Karbstein, 2017). Importantly, the loss of this RP has substantial effects on translation: eS26‐containing ribosomes interact with the Kozak sequence to drive rapid translation of mRNAs with cytoplasmic translation‐associated functions, but those depleted for the RP conversely favour translation of mRNAs with cell cycle, DNA repair and stress‐response functions (Ferretti et al., 2017). In another recent study, between 15 and 20% of 80S ribosomes were shown using cryo‐electron microscopy to lack the RPs eS1 (Rps1) and uL16 (Rpl10) (Sun et al., 2018). Furthermore, the proportion of ribosomes lacking these RPs increases to >30% following a switch in growth medium from glucose to glycerol (Sun et al., 2018).

A number of the RPs discussed above, including eS26 and eS1, cluster around the mRNA exit channel (Figure 3a), suggesting that this region acts as a regulatory hub for modulating protein synthesis. In addition, uS7 (Rps5) is located in this region, and contacts mRNAs to promote efficient translation initiation and discrimination of cognate over near‐cognate start codons (Visweswaraiah, Pittman, Dever, & Hinnebusch, 2015). On the other hand, uS3 (Rps3) plays a related role at the mRNA entry channel on the opposite side and also promotes accurate initiation (Dong et al., 2017). Although no regulatory role for either uS7 nor uS3 has been proposed, they contribute to the rich network of mRNA‐protein interactions in the vicinity of the mRNA channel. It appears that the association of some of these mRNA–RP interactions can be modulated during changing conditions, providing a means to directly and rapidly alter ribosomal interactions with mRNAs. The loss of certain RPs can therefore act as a switch to activate or deactivate specific translational programs and adapt the proteome upon exposure to stress.

## 6 CONCLUSIONS

The role of specific mechanisms for translational regulation is now widely appreciated, as technological advances in mass spectrometry and RNA sequencing have elucidated relatively subtle aspects of global translational regulation. It is now apparent that mRNA sequence and structure elements greatly impact the influence that ‘global’ regulatory mechanisms have over them. Many of these effects are transduced by RNA‐binding proteins which recognize such elements in groups of related mRNAs to bring about coordinated changes in translation. Just as RBPs bind to many mRNAs, a single mRNA may contain elements that allow it to bind numerous different RBPs. A huge network of mRNA–protein interactions therefore underpins mRNA fate, and the translational outcome of a change in conditions will be the sum of all the different interactions each mRNA makes, rather than a binary switch controlled by only one factor. More recent observations that some core ribosomal proteins interact themselves with specific mRNAs to promote their translation add further complexity to this network and further blur the line between RPs and RBPs, as RBPs can also be seen as accessory ribosomal components. Recent studies directly demonstrating stoichiometric differences between core RPs in actively translating ribosomes are particularly notable, providing clear evidence that core ribosomes do not all possess the same intrinsic translational capability. Furthermore, the observation that ribosomal composition can differ during stress conditions suggests the exciting possibility that ribosomes themselves can be remodelled to regulate translation and ensure cell survival. Much future work will be required to define the extent of ribosome structural heterogeneity in terms of differential stoichiometry, modifications and paralogue incorporation, as well as whether this may differ during changing cellular conditions. It will also be interesting to address the mechanisms by which ribosomes may become specialized and potentially remodelled. Of course, it will be important to focus on the functional impact of any differences in ribosomal composition to understand how different ribosomal sub‐populations contribute towards cell translational regulation and cell survival.

## CONFLICT OF INTEREST

The authors declare no conflicts of interest.

## FUNDING

Work in the Pavitt laboratory is funded by grants from the BBSRC (BB/M006565/1 and BB/N014049/1). R.A.C. is supported by a BBSRC Doctoral Training Program studentship to the University of Manchester (BB/M011208/1).
